# Palghat gap reveals presence of two diverged populations of Nilgiri tahr (*Nilgiritragus hylocrius*) in Western Ghats, India

**DOI:** 10.1080/23802359.2018.1436990

**Published:** 2018-02-15

**Authors:** Bheem Dutt Joshi, Rakesh Matura, Predit M. A., Rahul De, Bivash Pandav, Vipin Sharma, Parag Nigam, Surendra Prakash Goyal

**Affiliations:** Wildlife Institute of India, Dehradun, Uttakrakhand, India

**Keywords:** Low genetic diversity, population divergence, bottleneck, mtDNA, cytochrome b

## Abstract

Genetic analysis is an important tool in understanding population structure, genetic diversity, and phylogenetics of endangered species likely to be affected by microevolution and anthropogenic factors. Western Ghats landscape is one of the identified biodiversity hotspots in India, and micro-evolutionary processes are observed in this landscape due to the presence of the gaps in the mountain ranges. Nilgiri tahr is endemic to and distributed in this landscape while very little is known about genetic characteristics, population structure and impact of these gaps on the species. In the present study, two different populations of Nilgiri tahr from the north (NPG) and south (SPG) of Palghat gap (PG) were studied using the cytochrome b gene (Cyt b; 310 bp) of mtDNA genome in the Western Ghats, India. Two variable sites were observed in the Cyt b fragment while the mean pairwise genetic distance between these two populations was 0.007. All the samples phylogenetically clustered in either north or south of PG. The presence of shallow divergence indicates the presence of suitable habitat in past which may have facilitated movement between NPG and SPG. A subsequent change in Paleo-climatic conditions and gradual formation of PG may have resulted in population diversification during the Pleistocene. Besides, Forensically Informative Nucleotide Sequence (FINS) observed would help in geo-assigning any individual from NPG or SPG to understand the likely influences on population demography due to poaching.

## Introduction

Nilgiri tahr is endemic to Western Ghats, India (Schaller 1970), and has recently been shifted to the new genus *Nilgiritragus hylocrius* (Ropiquet and Hassanin 2005) from *Hemitragus*. Based on the molecular phylogeny, Nilgiri tahr is considered the sister group of the *Ovis* and therefore transferred to a new genus (Ropiquet and Hassanin 2005). Most of the Nilgiri tahr populations are fragmented and restricted to the long narrow stretch of 400 km between 11°30′ N to 8°20′ N in the Western Ghats (Schaller 1970; Davidar 1978). The Nilgiri tahr is a resident of montane grasslands with rocky cliffs at elevations of around 300–2600 m above mean sea level (Abraham et al. 2006; Predit et al. 2015). The populations of Nilgiri tahr have drastically decreased due to poaching, habitat degradation and other human activity, and therefore assigned as an ‘endangered’ species in the International Union for Conservation of Nature (IUCN) red list as well as in Schedule I under Indian Wildlife (Protection) Act 1972. However, population and conservation status have been studied (Chandran 1980; Wilson 1980; Rice 1984; Abraham et al. 2006; Predit et al. 2015) and the reasons for local distribution of the Nilgiri tahr are still limited in the areas of Western Ghats due to its preference for a habitat that is predominantly of grasslands. Identifying conservation units (CUs), management units (MUs), and evolutionarily significant units (ESUs) across the range of the species has been a key issue in effective conservation planning of species to retain the genetic diversity. However, no genetic assessment for Nilgiri tahr is available so far except for a study on cross amplification of nuclear markers from the Eravikulam National Park population (Luis et al. 2016). Out of the different gaps observed along the Western Ghats, Palghat gap (PG), which is 30–40 km wide, is a major biogeographic barrier for several species (Subramanyam and Nayar 1974; Ali and Ripley 1987). Recent molecular studies suggest PG is one of the drivers for diversification of various taxa, viz. the Asian elephant, the lion-tailed macaque, bush frogs, and birds e.g. white-bellied shortwing (Vidya et al. 2005; Robin et al. 2010; Vijayakumar et al. 2016) and next to the PG there is another gap present in south of Western Ghats of 7 km wide called Shencottah Gap (Ali and Ripley 1987). Therefore, the present study aims to perform a genetic assessment of Nilgiri tahr in the current distribution range of the species in the Western Ghats to identify any divergence between the populations caused by PG. For this, we used cytochrome b (Cyt b) of mtDNA genome to understand phylogeography and genetic diversity of Nilgiri tahr across the north and south of PG. The data on population genetics thus obtained would also help in the field of wildlife forensics for the detection and determination of the poaching hotspots in future as Nilgiri tahr has often been reported to be poached.

## Materials and methods

**Figure 1.**
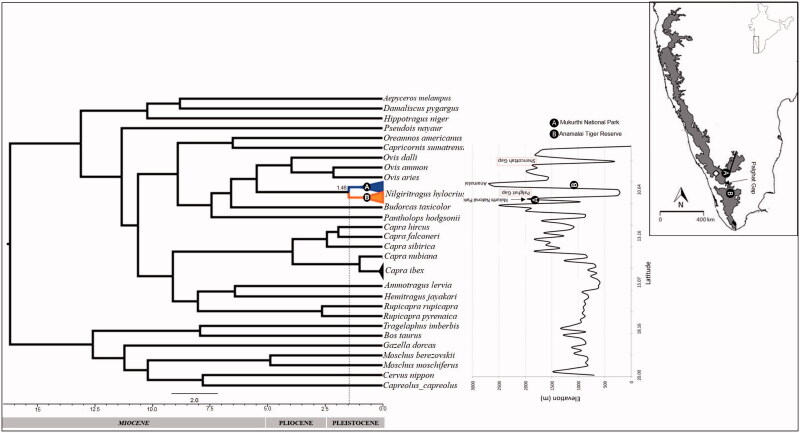
Phylogenetic tree (Bayesian) showing a divergence between two populations (North (A) and South (B) of Palghat Gap) of Nilgiri tahr in the Western Ghats, India (elevation profile adapted from Robin et al. 2010).

Faecal pellets were collected from North and South of PG from Mukurthi National Park (MNP) and Anamalai Tiger Reserve (ATR) respectively in the Western Ghats (Figure 1). These pellets were air dried and stored in silica gel covered by clean Kim-wipes (Kimberley-Clark, Irving, TX) to separate the desiccant from the pellets. Genomic DNA from samples was extracted using Qiagen DNeasy Stool DNA extraction kit (Qiagen, Hilden, Germany) as per manufacturer specified protocols with minor modifications where samples were incubated at 60 °C in a dedicated facility for faecal DNA extraction. The quantity and quality of the extracted DNA were estimated on 0.8% agarose gel stained with ethidium bromide. Polymerase chain reaction (PCR) was used to amplify the mitochondrial part of universal Cyt b (381 bp) gene fragment (Helm-Bychowski and Cracraft 1993). PCR was performed in a reaction mixture of 25 μl containing, 10 μl 2 × PCR master mix (Thermo Fisher Scientific, Waltham, MA), 4 μl template DNA, 1 μl primers (0.2 μM each), and 10 μl nuclease-free water. The amplification conditions were 94 °C for 5 min followed by 35 cycles at 94 °C for 30 s, 53 °C for 45 s, and 72 °C for 45 s, and a final extension was done at 72 °C for 10 min. The success of PCR amplification was verified using 2% agarose gel electrophoresis. The amplified products were then processed for cycle sequencing PCR using the Big Dye Terminator Cycle Sequencing Kit version 3.1 (Applied Biosystems, Foster City, CA) and were sequenced on an Applied Biosystems ABI 3500 Genetic Analyzer (Foster City, CA).

### Data analysis

Each Cyt b sequence was manually scrutinized using SEQUENCHER 4.8 (Gene Codes Corporation, Ann Arbor, MI). Total 310 bp long nucleotides were found suitable for the analysis after validating the sequences. Clean electropherograms and high-quality value (*Q* > 20) were considered for detecting mutation. Multiple sequence alignments were performed using the CLUSTAL W algorithm implemented in BIOEDIT version 7.0.5.33 (Hall 1999). The percent sequence divergence between clusters was calculated using Kimura’s two-parameter (K2P) method in MEGA version 6 (Tamura et al. 2013). Phylogenetic trees were reconstructed using different statistical methods, e.g. neighbour-joining (NJ), maximum likelihood (ML), and maximum parsimony (MP) implemented in the MEGA version 6 (Tamura et al. 2013). For distance and likelihood-based trees, we used a model of nucleotide substitution estimated in Modeltest 3.6 (Nylander 2004), and HKY model was used for the analysis based on the Akaike information criterion (AIC) value. Confidence in the estimated relationships was determined by calculating bootstrap values with 1000 replicates. Bayesian inferences (BIs) were conducted in BEAST v. 2.1.3 (Bouckaert et al. 2014). For the molecular clock rate, we used a normal prior and calibrated with the spilt between the Bovidae and Moschidae with mean divergence times of 16 Ma. We placed a standard deviation on the rate equal to 10% of the mean to account for variation and uncertainty in the rate. Dating analyses were performed for 20 million generations while sampling every 1000th tree, and the first 10% of trees sampled were treated as burn-in, and FigTree v.1.3.1 (Rambaut 2009) was used to display and summarize annotated phylogenetic trees yielded by BEAST.

## Results and discussion

### Sequence variability, genetic diversity, and demographic history

A total of 26 samples were sequenced from both the populations and two variable sites were found. Both the point mutations are transition A–G at the position of 14456 and 14472. Within the populations, no variable sites were observed. The mean pairwise genetic distance between the populations was 0.007 which is within the range of intra-species sequence divergence (Tobe et al. 2010). The overall nucleotide and haplotype diversity observed were 0.00337 and 0.520 (Table 1). Due to lack of within-population variable sites, diversity indices were not obtained within populations. We also compared the haplotype and nucleotide diversity of Nilgiri tahr with other Bovidae species and found a moderate level of nucleotide and haplotype diversity with the different mitochondrial genes (Table 1). We compared our data-sets with the different sets of genes in two species showing low genetic diversity comparable to Nilgiri tahr, viz. *Gazella arabica acacia* (Hd, 0.327; π 0.0051) and *Ovis ammon* (π 0.0054). The explanation provided for the low genetic diversity in *Ovis ammon* was recent population divergence (Tserenbataa et al. 2004), whereas *Gazella arabica acacia* has been found to have gone through population bottleneck being restricted to a small area (Hadas et al. 2015). In Nilgiri tahr, *F*_ST_ value between the populations (*F*_ST_=1) suggests there is no sharing of genetic material (Table 1). These results have also been supported by the characteristic of two different haplotypes and the fact that no haplotypes have been shared between both the populations of north and south of PG. Both Tajima’s *D* and Fu’s *Fs* test were found to be positive (Table 1) and statistically significant (*p*<.05) in the combined data. This indicates strong population subdivision, loss of rare alleles or population bottleneck (Hudson et al. 1987; Rogers and Harpending 1992; Fu 1997).

**Table 1.** Observed diversity and neutrality indices for the Nilgiri tahr population in the Western Ghats, India in comparison with other species of Bovidae.

Species	*N*	*V*	*F*_ST_	SD	Hd	π	Tajima’s *D*	Fu’s *F*s	Gene (bp)	Source
*Hemitragus hylocrius*	26	2	1.000; *p* = .000	0.007	0.520	0.00337	2.10822; *p* < .05	3.337	Cytb (381)	Present study
*Gazella subgutturosa*	102	–	0.57887	0.003	0.855	0.00224	–2.16917; *p* < .05	–41.95	Cytb (1140)	Tanchenge et al. (2016)
*Gazella dorcas*	28	10	–	–	0.802	0.01590	–	–	Cytb (790)	Hadas et al. (2015)
*Gazella gazelle*	54	21	–	–	0.937	0.00470	–	–	Cytb (790)	Hadas et al. (2015)
*Gazella arabica acacia*	11	2	–	–	0.327	0.00510	–	–	Cytb (790)	Hadas et al. (2015)
*Pseudois nayaur*	31		–		0.998 (0.009)	0.023 (0.001)	–	–	Cytb (1140)	Zeng et al. (2008)
*Procapra picticaudata*		113	–	–	0.88	0.060	–		Cyt b (425)	Zhang and Jiang (2006)
		46	–	–	0.975	0.081	0.043(0.506)	0.971 (0.712)	CR (570–720)	
*Procapra picticaudata*	51	–	–	–	0.97560.014	0.10560.01	0.377 (0.1)	0.971 (0.712)	CR (580)	Zhang et al. (2013)
*Pantholops hodgsonii*	118	–	–	–	0.99160.003	0.02560.10	0.971 (0.712)	224.08 (0.001)	CR (580)	
*Capricornis swinhoei*	–	90	–	–	0.930	0.02830	0.73740	–	CR (1014–1096)	Liu et al. (2013)
*Capricornis sumatraensis*	–	78	–	–	0.895	0.02490	0.22641	–		
*Capricornis crispus*	–	71	–	–	0.983	0.01760	–0.31634	–		
*Pseudois nayaur*	–	–	–	–	0.990	–	–1.39364; *p* > .10	–3.077; *p*<.01	Cyt b/CR (1694)	Tan et al. (2012)
*Pseudois nayaur*	31		–		0.998 (0.009	0.023 (0.001)	–	–	Cytb (1140)	Zeng et al. (2008)
	–	–	–	–	0.0993 (0.081)	0.108 (0.002)	–	–	CR (1227)	
*Ovis ammon*	–	–	–	–	–	0.0054	–	–	ND5 (556)	Tserenbataa et al. (2004)

Values in the parenthesis are base pair size of the mitochondrial region used.

*N*: number of sequences; *V*: number of variable sites; *F*_ST_: population differentiation; Hd: haplotype diversity; π: nucleotide diversity; SD: sequence divergence; *D*: Tajima’s test statistic.

### Phylogenetic, molecular dating, and diversification

In the phylogenetic tree, Nilgiri tahr populations cluster into two reciprocally monophyletic clades, which correspond to north and south of PG (Figure 1). However, the presence of long branches of Nilgiri tahr divergence from the *Ovis* probably indicates ancestor of this species was widely distributed across the Western Ghats (Figure 1). The divergence time estimates using fossils calibration, the point of the split between the Moschidae and Bovidae estimates the divergence between the populations of north and south of PG to be around 1.46 (95% highest posterior density; 0.80–2.34) million year ago (Ma). The presence of two diverse populations in the north and south of PG indicates that this gap may have played some role in population divergence. However, Nilgiri tahr is a less studied animal, and till now no evidence for the population divergence has been reported except the phylogenetic study confirmed Nilgiri tahr as the sister group of *Ovis* with the divergence time range from *Ovis* around 2.7–5.2 Ma (Ropiquet and Hassanin 2005). However, the origin of PG is debated and dated around 500 Ma ago (Soman et al. 1990) and is earlier than the reported divergence time for most of the species in the Western Ghats. Published studies on various taxa reveals that PG has been one of the prominent barriers to gene flow in the Asian elephants (Vidya et al. 2005), lion-tailed macaques (Ram et al. 2015), birds, e.g. white-bellied shortwing (*Brachypteryx major*) (Robin et al. 2010) and 10 other species (Robin et al. 2015), Nyctibatrachidae family of frogs (Van-Bocxlaer et al. 2012) and bush frogs (Vijayakumar et al. 2016) in the Western Ghats. PG also acts as a barrier for the gene flow in the plant species like *Eurya nitida* (Bahulikar et al. 2004) and *Gaultheria fragrantissima* (Apte et al. 2006). In this context, the divergence time estimated for different species varies from 0.29 Ma in the Asian elephant (Vidya et al. 2005), 2.4 Ma for lion-tailed macaques (Ram et al. 2015) to 2.5 Ma in the bush frog (Vijayakumar et al. 2016) and 40 Ma for the frog species of family Nyctibatrachidae (Van-Bocxlaer et al. 2012). Fish species of genus *Puntius* (red line barb) shows the greater divergence time 56 Ma than these observed for these taxa in north and south of PG (John et al. 2013). The divergence time obtained for Nilgiri tahr (1.46 Ma) is older than that observed for the elephant (0.29 Ma) but within the range of lion-tailed macaque and bush frogs (2.4–2.5 Ma).

PG acts as the potential barrier in spite of land connectivity across the 40 km wide gap. However, no extensive survey on palaeobotany is available which may elucidate how this gap acts as the barrier. Moreover, studies on the bush frog (Vijayakumar et al. 2016), Caecilians (Gower et al. 2007) and the shallow divergence in Nilgiri tahr as discussed in this study do not highlight PG as a significant barrier. In support of this, literature also reveals that PG has gradually formed by the differential uplift of the plateau on both sides and further erosion cycles due to the fluvial actions of westerly flowing streams (Rao and Srinivasan 1980; Medlicott and Blanford 1983). A detailed intra-specific phylogeographic study of different taxa is needed to identify the cause of the phenomenon (Joshi and Karanth 2013). Moreover, high human activity and loss of forest connectivity have also been considered as one of the cause for population differentiation (Vidya et al. 2005; Robin et al. 2015) though divergence time estimates reported are older than the anthropogenic activities, only supporting the theory of the Pleistocene diversification. Pleistocene climatic oscillations have been suggested to be the cause of the diversification and disjunct distribution of species in different landscapes (Clemens et al. 1991; McClymont et al. 2013), which is in accordance with the divergence time of the species studied so far across the north and south of PG in the Western Ghats.

### Conservation implication

The mitochondrial marker (Cyt b) used in the current study suggests the presence of reciprocally monophyletic clades in Nilgiri populations of north and south of PG in the Western Ghats. The literature reveals that the northern parts have been relatively drier than the southern Western Ghats during the Pleistocene and acted as a refugium for the species (Sukumar et al. 1993). Nilgiri and Annamalai regions in the Western Ghats display a phytogeographic barrier to have different floristic compositions (Subramanyam and Nayar 1974; Vidya et al. 2005). Such spatial differences in plant species and moisture regimes in the habitat are known to result in intra-specific variations in wild herbivores. Kangas et al. (2016) reported the presence of intra-specific geographic variation in the shape of mandible across the range of moose (*Alces alces*) regarding attachment surfaces of the muscles that control biting and mastication as a response to food plant characteristics. Due to lack of information on such anatomical variations in Nilgiri tahr, we believe that such difference may exist to harvest nutrients differently from plant species in these two populations of dry and moist regions of Western Ghats. Therefore, we suggest both the populations of the Nilgiri tahr should also be managed as a separate MU for *in situ* conservation plans as well as being considered as separate entities for *ex situ* conservation strategies. Besides, we also reiterate that thorough studies on anatomical differences, microsatellite and other mitochondrial markers are required to understand the extent of population divergence and gene flow in Nilgiri tahr populations across the Western Ghats.
